# Supplementary material for: Identification of photoperiod-induced specific miRNAs in the adrenal glands of Sunite sheep (Ovis aries)
Source: Front Vet Sci. 2022 Jul 22;9:888207. doi: 10.3389/fvets.2022.888207 (PMC9354845; doi:10.3389/fvets.2022.888207)
Supplement: Supplementary file 1 [file Data_Sheet_1.ZIP › Supplementary file/miRDeep2 file.zip!/miRDeep2 file/Know_Structure/SP42A2/oar-mir-655.pdf]

miRBase precursor : oar-mir-655
Total read count : 2945
oar-miR-655-5p read count : 8
oar-miR-655-3p read count : 2937
remaining reads : 0

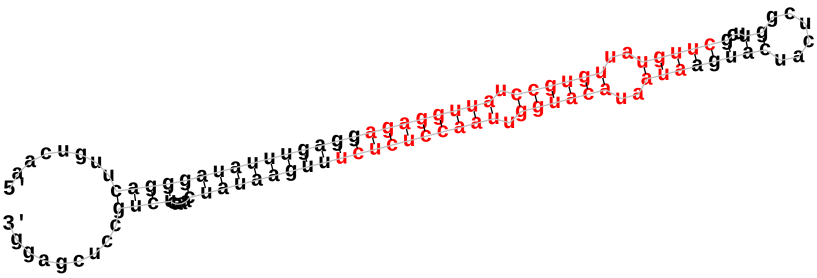

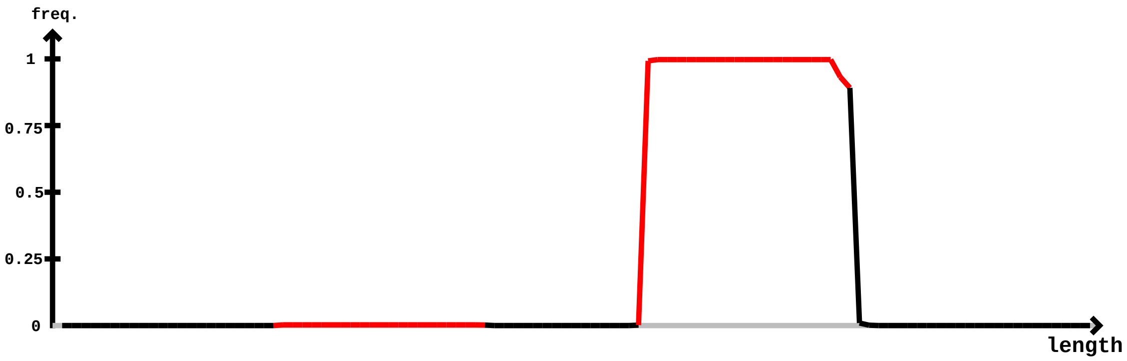

oar-miR-655-5p

oar-miR-655-3p

```
5'- aacuguucagggauauuugaggagagguuauccguguuauguucgcuuggcucaucaugaauaauacaugguuaaccucucuuugaauaucaaacucugccucgagg   -3'    exp
    ..........((((((((((((((((((.((((((..((((((..((......))))))))..)))))).))))))))))))))))))...(((......))).    reads    mm    sample
    ......................agagguuauccguguuauguu................................................................    2        0     seq
    ......................agagguuauccguguuauguuc...............................................................    6        0     seq
    ...........................................................aauaauacaugguuaaccucu...........................    5        0     seq
    ............................................................auaauacaugguuaaccucu...........................    186      0     seq
    ............................................................auaauacaugguuaaccucuc..........................    120      0     seq
    ............................................................auaauacaugguuaaccucucu.........................    2586     0     seq
    ............................................................auaauacaugguuaaccucucuu........................    22       0     seq
    ............................................................auaauacaugguuaaccucucuuu.......................    4        0     seq
    .............................................................uaauacaugguuaaccucu...........................    1        0     seq
    .............................................................uaauacaugguuaaccucucu.........................    11       0     seq
    .............................................................uaauacaugguuaaccucucuu........................    2        0     seq
```
